# Federated transfer learning for rare attack class detection in network intrusion detection systems

**DOI:** 10.1038/s41598-025-02068-x

**Published:** 2025-09-30

**Authors:** Chunduru Sri Abhijit, Y. Annie Jerusha, S. P. Syed Ibrahim, Vijay Varadharajan

**Affiliations:** 1 School of Computer Science and Engineering, Vellore Institute of Technology, Chennai Campus, Chennai, 600127 India; 2 Advanced Cyber Security Engineering Research Centre, The University of Newcastle, Callaghan, Australia

**Keywords:** Artificial intelligence (AI), Federated learning (FL), Few shot learning (FSL), Network intrusion detection system (NIDS), Technological innovation, Zero-day attacks, Rare classes, Computer science, Information technology, Scientific data

## Abstract

Federated learning (FL) offers a promising approach for training machine learning models with minimal data sharing, enhancing privacy and performance. However, building effective FL-based network intrusion detection systems (NIDS) remains challenging due to the need for large, diverse training datasets. Identifying rare attack types with limited instances is a persistent obstacle, and their detection is critical in cybersecurity. This research introduces a novel FL framework to address these challenges. By incorporating adaptive, personalized layers at the client level, the model reduces false alarm rates for zero-day attack types and improves the detection of rare classes. The model also leverages Transfer Learning (TL) to identify zero-day attacks, where client-specific gradients are collected and used to update a global model on the server side after multiple rounds of exposure to new data. The proposed sustainable framework aims to disseminate knowledge about rare attack types across clients through a server-based global model within the FL ecosystem. This study achieves two main objectives: (i) improving the detection of rare attack classes and (ii) identifying zero-day attacks in a NIDS context. Evaluations on the CSE-CICIDS-2018, Edge IIoT, and UNSW-NB 15 datasets, which encompass diverse class distributions, demonstrate that the proposed approach outperforms existing models in detecting and handling rare and novel attack types. The proposed model achieves 98.90% accuracy on CICIDS 2018, 98.70% on UNSW-NB 15, and 97.92% on Edge-IIoT, surpassing the FL-TL-CNN model by 2.78%, 1.51%, and 2.03%, respectively. These results highlight the effectiveness, robustness, and adaptability of the proposed approach in enhancing intrusion detection across heterogeneous network environments.

## Introduction

Based on a study by Aiyer et al.^1^, more than 80 percent of the threat groups determined in 2021 and more than 40 percent of the malware found were unknown to security researchers. These patterns indicate significant potential in a quickly changing world. We utilize Network Intrusion Detection Systems (NIDS) to identify any malicious network traffic that could compromise the confidentiality, integrity, and availability of information within a computer network. NIDS tools analyze incoming traffic to recognize any indications of a potential threat. In recent days, applying machine learning-based solutions^2^ to NIDS has been of prime interest among security researchers and specialists. The challenge of gathering a large amount of labeled data in a single location and the model’s poor performance on large volumes of data and high-class imbalance data make using these models challenging.

With the ability to extract high-level features from raw data, such as network traffic, deep learning-based NIDS models have consistently demonstrated superior performance as in^3^ to traditional NIDS models. The conventional methods include signature-based and rule-based systems, as seen in^4,5^. However, one of the biggest challenges with these models is the need for large amounts of labeled data for training, which can be difficult to obtain in real-world scenarios and is often prohibitively expensive. Despite these challenges, we cannot overlook the advantages of deep learning in NIDS.

One of the critical issues in NIDS is detecting rare classes, which represent attack categories that occur infrequently within network traffic. These classes often have significantly fewer instances than normal traffic or majority attack types, making it difficult for machine learning models to classify accurately. The challenge is further exacerbated by zero-day classes, referring to new and previously unseen attack types that exploit unknown vulnerabilities before detection mechanisms can recognize them. Since zero-day attacks have no prior labeled data, they are even more difficult to detect using traditional classification techniques. Rare class detection poses a significant challenge when dealing with large network datasets containing infrequent classes. To build a high-performance NIDS capable of unbiased detection across all classes, a strong emphasis is essential. Without adequate strategies for identifying rare classes, the model may fail to detect these less common but potentially critical threats. Addressing this imbalance ensures that the NIDS model remains robust, accurate, and effective in detecting a broad range of attack types, contributing to comprehensive network security.

Federated Learning (FL) is a method that enables different locations, or “clients,” to train a shared machine learning model without directly sharing sensitive data. Instead of sending raw data to a central server, each client trains the model on its data locally and only sends back updates (known as “gradients”)^6^. This way, the central server can build a global model by combining updates from multiple clients, protecting data privacy, and reducing data-sharing risks. This approach is beneficial for applications like NIDS, where gathering data from multiple sources (e.g., different network environments) can improve the model’s ability to detect threats. By leveraging data from a variety of sources, FL-based NIDS can potentially achieve higher accuracy without compromising privacy. However, detecting rare attack types with very few examples remains a challenge in federated setups, as the model may struggle to learn from these limited instances effectively.

Due to the dynamic nature of attacks and data distributions, current models may be outdated and unable to forecast new distributions accurately, resulting in incursions. Cyber hackers regularly develop new types of assaults. If current models do not receive training on these previously unseen/rare attack classes, they may fail to recognize the hostile agent, leading to a breach. When any client discovers a new unseen/rare class, it is crucial to update clients throughout the ecosystem promptly. Traditional federated learning solutions, such as Fedavg^7^ and FedProx^8^, use the same model architecture across clients for gradient aggregation, limiting client-side personalization. Dynamically changing attack classes necessitate the use of personalized layers. Therefore, we propose a unique, lightweight, personalized Transfer Learning (TL) architecture that can enhance the detection of new classes and simultaneously boost the performance of rare classes through transfer learning, thereby disseminating this knowledge to all clients.

We worked to improve the performance-to-cost ratio of federated learning with rare class detection and to find and report attacks that haven’t been seen before in the networks. These are our main contributions:

1. *Adaptive personalized federated averaging with client-specific layers*: The proposed novel model presents a federated learning framework that integrates adaptive, client-specific layers to enhance personalization and enable more tailored responses to local data variations. On the server side, it utilizes a selective averaging mechanism to effectively aggregate model updates across diverse clients. This dual approach not only boosts the detection capabilities for rare and unseen attack classes but also reduces false alarms, which is critical for efficient deployment in resource-constrained environments.
2. *Transfer learning and threshold-based detection for zero-day attacks* Our framework incorporates transfer learning along with a threshold-based detection mechanism to address the unique challenges posed by zero-day attacks. By enabling clients to test model performance on newly observed attack classes and to communicate relevant metrics to the server, the model can effectively adapt and expand its detection capabilities. This approach supports real-time, dynamic updates, enhancing the federated model’s ability to generalize to new and unknown threat types.
3. *Extensive experimentation and benchmarking* We conducted extensive experimentation on three widely recognized datasets, CSE-CICIDS-2018, Edge IIoT, and UNSW NB-15, which represent a range of cyberattack scenarios. The results show that the proposed framework consistently outperforms baseline models at finding new and rare attacks, with accurate classification results across all datasets. This showcases the model’s practical applicability and potential to improve NIDS in federated learning environments.

We organize the remainder of this paper as follows: “Materials and methods” section introduces the materials and methods used to construct the work. Section “Related work” shows the related work followed. Section “Proposed FL-TL model” delves into a detailed discussion of the proposed method, while “Experimental methodology” section encompasses the experimental methodology. Section “Experimental results” presents the results, while “Conclusion” section presents the conclusions.

## Materials and methods

### Localized learning

In the context of NIDS, localized learning involves training the system to detect and respond to threats specific to a particular network or environment. This is done by analyzing network traffic and identifying patterns and anomalies unique to that network. This learning methodology can improve the accuracy and effectiveness of intrusion detection and response and help reduce false positives and negatives. Networks are dynamic environments where traffic patterns can change rapidly due to factors such as new applications, software updates, or changes in user behavior. A localized NIDS needs to adapt quickly to these changes to maintain its effectiveness. However, this adaptability can be challenging to achieve without compromising detection accuracy. Consider a dataset *X* = $\{x_{1},x_{2},x_{3},...x_{N}\}$ where N is the number of data samples in the dataset and Y = {$y_{i}$} is a class label that each data sample possesses. Each data sample is mapped to a feature vector $v_{i}$ in a high-dimensional feature space. We use a classifier model (machine learning model) C to assign a label $y_{i}$ to a $x_{i}$ and shown as $y_{i} = C(v_{i}$).

### Centralized learning

Centralized learning in NIDS involves collecting network traffic data from multiple points in a network and sending it to a central location for analysis and decision-making. This technique allows for a more comprehensive network traffic analysis since data is collected from multiple points in the same network. This enables the system to detect threats that might not be apparent by analyzing data from a single point of view. Centralized learning can reduce false alarm rates^9^. However, there are also some drawbacks to centralized learning; one potential issue is that centralized learning typically relies on a central server or a centralized model that requires access to a large amount of data. In the learning scenarios of rare classes, where only a limited amount of data is available per class or task, this dependency on abundant data becomes a hindrance. Models may struggle to generalize effectively from limited data samples. A detailed explanation is given in the Appendix.

### Federated learning

Federated learning is a machine learning technique that allows a server and multiple clients to collaboratively train a shared model without exchanging raw data. In traditional FL-NIDS^10,11^, a central server collects shared network model weights and uses machine learning algorithms to analyze the data for potential security threats. This approach has several potential benefits, including improved privacy, reduced network bandwidth, and improved accuracy. This approach also addresses the few-shot challenge. The detailed explanation is given in Appendix.

### Transfer learning

Transfer learning^12–14^ in NIDS involves leveraging knowledge gained from one domain to improve the performance of intrusion detection in another domain. The model would yield better results if the domains were relatively similar. Here’s how transfer learning can be applied in NIDS: (*i*) Feature transfer: One can use transfer learning techniques to transfer feature representations learned from one dataset to another. For instance, you can transfer features learned from one network environment to another to enhance detection accuracy. (*ii*) Model transfer: We can fine-tune pre-trained models from related domains for intrusion detection tasks. Models trained on large-scale datasets, such as general network traffic or even natural language processing tasks, can capture generic patterns that are useful for detecting intrusions. We apply transfer learning in ensemble learning, domain adaptation, and many other fields.

Our proposed model uses a transfer learning methodology to improve the detection of rare classes containing very few shots. Detailed explanation is given in Appendix.

### Personalized deep neural networks (DNN)

Personalized splitting is an emerging technique in deep neural networks that facilitates model personalization for clients with diverse data distributions, particularly within federated learning and other distributed frameworks. This method addresses the challenge of model heterogeneity by partitioning the model into shared and personalized components. Specifically, the initial layers of the model are shared across clients, capturing generalizable features and leveraging aggregated client data to build a common representation. For instance, in a convolutional neural network (CNN), the shared layers might extract basic textures and edges, features that are common across various types of images. Conversely, the later layers of the network are designated as personalized layers, which are fine-tuned on each client’s unique data distribution. This structure enables the model to adapt to the specific requirements of individual clients by capturing more contextual or unique class information relevant to each client. Training in personalized splitting involves each client updating only their personalized layers based on local data, while the shared layers are periodically aggregated across clients. This split training structure enables collaborative learning in the shared layers while preserving individual client performance in the personalized layers. In certain implementations, hybrid update strategies are adopted where shared layers are aggregated at fixed intervals, whereas personalized layers are continuously updated. Alternatively, weighted averaging of the shared layers can be employed to balance global and local learning, allowing each client to maintain self-sustaining personalized layers.

The model parameters can be mathematically represented as $\theta = [\theta _s, \theta _p]$, where $\theta _s$ denotes the shared parameters, and $\theta _{p_k}$ represents the personalized parameters for each client *k*. Each client *k* minimizes a local loss function $L_k(\theta _s, \theta _{p_k})$, updating their personalized parameters independently. Post-training, only the shared parameters $\theta _s$ are aggregated across clients, whereas the personalized parameters $\theta _{p_k}$ remain specific to each client. The aggregation of shared parameters across *K* clients can be expressed as:

1 $$\begin{aligned} \theta _s^{(t+1)} = \sum _{k=1}^{K} \frac{|D_k|}{|D|} \theta _s^{(t)} \end{aligned}$$

### Datasets

The three datasets—CSE-CICIDS 2018, UNSW-NB15, and Edge-IIoT-offer varied attack scenarios and data distributions, effectively simulating real-world federated learning environments for intrusion detection. Tables 1, 2, 3 shows a detailed dataset split of CSE-CICIDS 2018, UNSW-NB15, and Edge-IIoT datasets, respectively.

**Table 1:** Attack data distribution across clients and test dataset of CSE-CICIDS 2018 dataset.

Attacks	Client 1	Client 2	Client 3	Client 4	Test dataset
Benign	100000	100000	100000	100000	100000
Bot	15000	15000	12000	20000	4500
DDoS attack-HOIC	0	100000	100000	100000	10000
DDoS attacks-LOIC-HTTP	61000	0	70000	75000	10000
DoS attacks-Hulk	55000	60000	0	45000	7500
DoS attacks-SlowHttpTest	25000	30000	15000	0	4500

**Table 2:** Attack data distribution across clients and test dataset of UNSW-NB15 dataset.

Attacks	Client 1	Client 2	Client 3	Client 4	Test dataset
Normal	100000	100000	100000	100000	100000
Generic	50000	35000	45000	10000	15000
Exploits	0	4000	5000	3000	5000
Fuzzers	3000	0	2000	2500	4000
Dos	2000	2000	0	2000	2000
Reconnaissance	2000	2000	2000	0	2000

**Table 3:** Attack data distribution across clients and test dataset of Edge-IIoT dataset.

Attacks	Client 1	Client 2	Client 3	Client 4	Test dataset
Normal	100000	100000	100000	100000	100000
Ddos-UDP	20000	35000	15000	10000	15000
Ddos-ICMP	0	3500	6000	13000	5000
SQL Injection	7000	0	12000	2500	4000
Ddos-TCP	9000	12000	0	4000	2000
Password	14000	13000	3000	0	2000

In the CSE-CICIDS 2018 dataset, clients observe “Benign” traffic alongside attacks like HOIC and LOIC-HTTP. Some clients miss specific attack types (e.g., “DDoS attack-HOIC” or “DoS attacks-Hulk”), reflecting the non-uniform data common in decentralized networks.

The UNSW-NB15^15^ dataset includes “Normal” and attacks such as “Generic,” “Exploits,” “Fuzzers,” “Dos,” and “Reconnaissance,” but not all attack types appear across clients. This heterogeneity, with some clients lacking “Exploits” or “Fuzzers,” mirrors realistic data constraints in federated setups.

In the Edge-IIoT^16^ dataset, the data spans “Normal” traffic, various DDoS types, SQL Injection, and Password attacks, with certain classes, such as “Ddos-TCP” or “Password,” absent in specific clients. This setup is particularly relevant in federated learning, where clients often have incomplete, heterogeneous data.

## Related work

This section examines key research papers focused on evaluating NIDS for their effectiveness in detecting rare and unseen classes in FL.

Deep learning-based intrusion detection systems (IDS) have gained significant attention in recent years due to their ability to extract high-dimensional features and improve detection accuracy. A deep learning-based intrusion detection framework presented in^17,18^ incorporates advanced feature extraction, optimization, and generative models to enhance attack detection and classification. The approach effectively addresses data imbalance and improves accuracy across multiple cybersecurity datasets, outperforming traditional methods. However, the framework may face scalability challenges with real-time intrusion detection in large-scale networks.

In the domain of machine learning-based approaches, Mehedi et al.^19^ introduced an IoT-specific Deep Transfer Learning (DTL) IDS that identifies normal and attack scenarios with minimal labeled data via attribute selection. Their approach, based on a reliable DTL-based ResNet model, achieved 87% accuracy, outperforming benchmark models across heterogeneous IoT networks. Additionally, Fan et al.^20^ developed IoTDefender, which integrates federated learning and transfer learning. The model enhances intrusion detection by transferring knowledge across clients using a public dataset. Their experiments on the CICIDS2017 dataset showed a 3.13% improvement in intrusion detection accuracy over traditional ML approaches. Ye et al.^21^ developed a rare class detection model using the Aviator technique for few-shot learning, a methodology aimed at constructing an unbiased detection model by considering task context during initialization. Task-specific initialization speeds up model optimization for high-quality solutions. Zhao et al.^22^ analyzed the impact of non-IID data on the performance of machine learning models, such as convolutional neural networks (CNNs) for classification tasks, observing a decline in model accuracy. They demonstrated that using a small subset of client data could improve performance. However, this approach introduced a drawback, as transmitting the subset of data led to information leakage about the training data. Abhijit et al.^23^ proposed a NIDS methodology using an FL framework to enhance IoT security on the UNSW-NB15 dataset. Feature selection is performed based on correlation, and five machine learning models-Logistic Regression, K-Nearest Neighbors, Random Forest, Decision Tree, and Artificial Neural Network-are implemented. Random Forest achieved the highest performance for both binary and multi-class classifications. However, the centralized ML approach raises privacy concerns and incurs high data transfer costs. The proposed FL approach addresses these limitations by training models locally on each IoT device, sharing only model weights, thereby preserving data privacy and reducing data transmission overhead. FL presents challenges, including model convergence issues and communication efficiency across decentralized devices.

Hospedales et al.^24^ present a unified active learning model that jointly addresses rare class discovery and classification by dynamically adjusting query criteria. It combines active discovery for quick identification of new categories with active learning to minimize labeling effort, using a classifier-switching mechanism between generative and discriminative models. Extensive UCI and vision datasets evaluations demonstrate the model’s advantage over existing methods. However, balancing discovery and classification remains challenging, especially in domains with extremely scarce rare classes, which may limit efficiency.

Federated learning (FL) has been increasingly explored to enhance IDS while preserving data privacy. Zhao Ruijie et al.^25^ proposed a semi-supervised FL approach for intrusion detection in IoT environments, addressing key limitations of existing methods. Their approach employs knowledge distillation to leverage unlabeled data and utilizes CNNs for deep feature extraction, both as classifier and discriminator networks. Despite these advancements, their method’s reliance on the quality of unlabeled data may impact performance. Agrawal et al.^26^ introduced F-BIDS, a blending approach combining deep learning and FL to efficiently assess and generalize massive volumes of sensitive data. Their experiments on Edge-IIoTset and InSDN demonstrated that FL-based methods reduce privacy concerns while achieving comparable performance to centralized ML models.

Expanding on federated learning methodologies, Khan et al.^27^ proposed a collaborative Simple Recurrent Unit (SRU) network with dynamic behavior aggregation for IoT-based medical networks (IoMT). Their model combines behavior-based and anomaly-based IDS within an FL architecture, ensuring privacy-preserved collaborative training. The system employs LIME for explainable decision-making and dynamically manages participating clients, reducing communication overhead. However, excluding underperforming clients could lead to biased global model updates. A similar work,^28^, introduced Federated-SRU for IoT-augmented Industrial Control Systems (ICS), leveraging skip connections and a bidirectional architecture to address vanishing gradients and computational costs in RNN variants. While the model maintains privacy and scalability, imbalanced data distributions across clients remain challenging.

Yang et al.^29^ introduced a novel adversarial attack methodology combining adversarial sample generation with poisoning attacks in the context of adversarial learning and FL-based attack mitigation. Their WM-GAN model utilizes a federated global model to generate adversarial samples and pre-training during attack preparation. Including a vulnerability dataset enhances attack invisibility, but embedding malicious features in federated environments remains challenging. Mao et al.^30^ proposed a federated knowledge transfer framework using model segmentation distillation and consistency-constrained loss to improve global and local models in high-heterogeneity settings. However, mitigating performance degradation due to non-IID data across clients while minimizing computational overhead remains an open problem.

Finally, recent works have focused on federated transfer learning-based models. Ji et al.^31^ proposed FTLCNN, an IDS that leverages federal transfer learning and convolutional neural networks to address challenges such as sample shortages, data asymmetry, and uneven distributions. Federal transfer learning enables learning models without exposing sensitive training data, ensuring data privacy and mitigating institutional data-sharing limitations. Their experiments on the UNSW-NB15 dataset demonstrated improved false positive detection compared to benchmark models. Similarly, Rodríguez et al.^32^ proposed a transfer learning-based IDS for IoT networks, focusing on zero-day attacks. Their method trains CNNs on one dataset before applying them to another, enhancing detection accuracy and reducing false positives. The Fed-Inforce-Fusion model (Fed-InF)^33^ integrates reinforcement learning with FL to detect complex cyber-attacks such as DDoS and ransomware in IoMT networks. The dynamic fusion strategy enhances detection accuracy while minimizing communication overhead, though scalability to large-scale heterogeneous networks presents challenges. Rajesh et al.^34^ used a Federated Transfer Learning (FTL) approach for IIoT intrusion detection, where a combinational neural network splits IoT data between clients and a server to create models. Client model weights are periodically aggregated to update the central server, enhancing privacy and improving performance over traditional ML. This setup shows high efficiency in detecting intrusions across IIoT networks. However, frequent weight updates introduce communication overhead, potentially impacting real-time detection in large-scale networks.

Wu et al.^35^ developed the FETLSVMP method that combines federated and transfer learning with support vector machines (SVM) for privacy-preserving intrusion detection across organizations. It aggregates distributed data through federated learning, while transfer SVM adapts to varying data distributions, building personalized models for improved detection. This approach enhances performance, especially on small samples and new intrusions. However, using SVM may limit scalability in high-dimensional data environments, impacting efficiency with increased data complexity.

Zhan et al.^36^ proposed a bidirectional federated transfer learning method that jointly leverages data from multiple Operational Technology (OT) and IoT domains for improved intrusion detection in IIoT environments. The model addresses diverse attack types and feature variations by integrating heterogeneous data from datasets like Edge-IIoTset, WUSTL-IIOT-2021, and Electra (Modbus). The federated approach allows decentralized learning while maintaining data privacy, and the transfer learning component enables adaptation to domain-specific packet characteristics. However, data imbalance and computational complexity across domains may impact the model’s performance, which could affect real-time applicability.

Wang et al.^37^ introduced an FTL framework aimed at diagnosing insulation defects in gas-insulated switchgear (GIS) while ensuring data privacy. The methodology leverages federated adversarial learning to achieve domain adaptation, allowing clients to collaboratively develop models without sharing data. A federated minimax (FedMM) algorithm is employed for global model aggregation, addressing gradient drift due to sample imbalance and enhancing accuracy. Experimental results indicate the approach performs robustly, especially with unbalanced small samples. However, challenges remain regarding model complexity and the potential variability in data quality across different clients, which may impact generalizability.

Friha et al.^38^ introduced the Federated Learning-based Intrusion Detection System (FELIDS) to secure agricultural IoT infrastructures. The methodology emphasizes local learning, allowing devices to share only model updates with a central aggregation server to protect data privacy. To enhance attack detection, FELIDS utilizes three deep learning classifiers-deep neural networks, convolutional neural networks, and recurrent neural networks. Performance evaluations on datasets like CSE-CIC-IDS2018, MQTTset, and InSDN reveal that FELIDS outperforms traditional centralized ML approaches in accuracy and privacy protection. However, challenges may arise in model generalization due to the reliance on local data and potential variability in client data distribution.

Sarhan et al.^39^ present a collaborative cyber threat intelligence sharing scheme to improve ML-based NIDS across multiple organizations. The methodology focuses on converting heterogeneous network data samples into a standard format, facilitating the extraction of meaningful patterns while employing an FL approach to preserve data privacy. The framework was evaluated using two NetFlow datasets, NF-UNSW-NB15-v2 and NF-BoT-IoT-v2, alongside comparisons to centralized and localized training methods. Results indicate that the proposed scheme efficiently classifies benign and intrusive traffic types without inter-organizational data sharing. However, a potential limitation lies in the dependency on the quality and representativeness of the shared threat intelligence, which may vary across organizations.

Tabassum et al.^40^ introduce FEDGAN-IDS, a Federated Deep Learning Intrusion Detection System that utilizes Generative Adversarial Networks (GAN) for data augmentation to address the imbalance in rare classes during training. The methodology involves distributing the GAN network across various IoT devices, enabling them to act as classifiers and train on locally augmented data. The model’s performance is evaluated by comparing its convergence and accuracy against existing federated intrusion detection systems. Extensive experiments across multiple datasets demonstrate that FEDGAN-IDS outperforms traditional standalone intrusion detection systems, achieving faster convergence and enhanced accuracy. However, a limitation of this approach may be the dependence on the quality of the generated augmented data, which could influence the overall effectiveness of the model if not adequately managed.

Most of the aforementioned research employed transfer learning with federated learning to initialize global models or transfer weight knowledge across domains. Their objective was to enhance the identification of rare classes during training. However, conventional FL techniques may hinder the customization of training to individual client needs, limiting adaptability to dynamic class distributions. Additionally, challenges persist in data availability, instability in the adversarial generation, and handling imbalanced data distributions. Our primary contribution lies in detecting and classifying zero-day attacks while enhancing rare class detection with a few-shot learning approach during the testing phase. We achieve this by leveraging transfer knowledge and the Federated Personalized Layer Aggregation principle, enabling adaptive training of new attack classes and refining model weights for improved generalization across heterogeneous environments.

## Proposed FL-TL model

### Proposed model overview

The proposed model introduces a personalized FL framework for NIDS that aims to detect rare and zero-day attack classes more effectively. This approach leverages personalized layers on the client side and TL to adapt the global model to each client’s unique data distribution, enhancing detection performance for rare classes while preserving privacy. This framework effectively combines federated learning with adaptive personalization and transfer learning to create a robust NIDS. It addresses the challenges of rare and zero-day attack detection by:

1. Enhancing client models with personalized layers tailored to specific data distributions.
2. Reducing communication through threshold-based “N” round updates.
3. Maintain a representative set of exemplars for the accurate classification of new data.

Personalized Federated Learning Architecture:

The model architecture is divided into two main sections: the shared standard layers and the personalized layers. Each client has its personalized layers tailored to its specific data distribution. These personalized layers focus on learning the unique patterns and rare attack characteristics of each client’s data, while the shared standard layers capture the general features common across clients. During training, only the shared layers are aggregated on the server, which minimizes the communication cost and maintains scalability. This selective sharing enables each client to retain a degree of customization while benefiting from the aggregated knowledge of other clients. The personalized layers, which are never shared with the server, allow each client to fine-tune its model based on local data characteristics, addressing client-specific attack patterns, including rare and unseen classes.

Transfer Learning with “N” Round Updates:

To minimize communication frequency, the model applies a transfer learning approach by synchronizing updates every “N” round rather than every round. In each “N” round, a threshold is calculated based on the change in performance metrics from previous rounds. If the accumulated difference surpasses a defined threshold, an update is sent to the global model on the server. This strategy conserves bandwidth and enhances efficiency by focusing on significant updates that contribute meaningful knowledge to the global model.

Exemplar-Based Global Model for Rare Class Detection:

The server maintains an exemplar set representing each class, with mean feature representations for each exemplar. These exemplars are updated periodically with client data, creating a balanced, representative set for effective new data classification. The global model computes the mean representation for each class based on these exemplars, which enables the model to generalize across diverse data sources. When new data arrives, it is classified by measuring the distance from each class mean. If the distance exceeds a predefined threshold, the data is considered to belong to a new class. This adaptive thresholding mechanism ensures the model remains responsive to novel attack patterns, facilitating effective zero-day attack detection.

Training and Classification:

During training, the global model iterates through multiple epochs, shuffling the exemplar set and updating weights based on cross-entropy loss. Each new class introduced by clients is processed using one-hot encoding and integrated into the server’s dataset, allowing the global model to incorporate novel attack types effectively. This setup enables the model to adapt to changes in class distributions across clients while preserving client-specific knowledge through personalized layers.

### Training clients with personalized layers

**Fig. 1:**
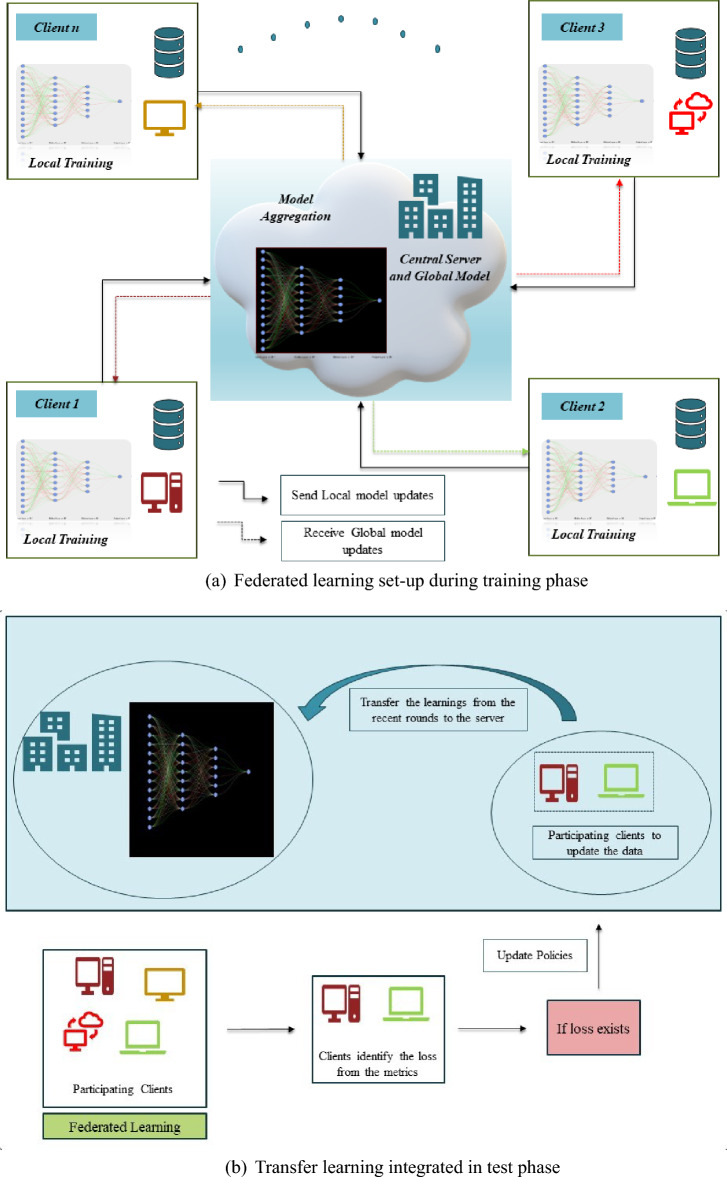
Proposed federated learning model with transfer learning.

As shown in Fig. 1, the proposed model guarantees that models are customized to meet individual requirements, resulting in enhanced performance on activities at the local level. Personalized Federated Averaging allows customizing models to match clients’ unique data distribution and preferences. It selectively transmits the model parameters pertinent to the central server rather than sending whole model updates. This minimizes the communication required, resulting in improved scalability and efficiency of federated learning. Personalized Federated Averaging can adjust to dynamic variations in the federated learning setting, such as the inclusion or exclusion of clients, alterations in data distributions, or modifications to privacy restrictions.

Since every single client would have different classes, the architecture used in the last layer would not be acceptable for federated average aggregation on the server. As a result, we decided to split the network into two distinct sections: the first is the standard layer that all clients share, and the second is the personalized layer that is only accessible to clients with customized layers. During the aggregation process, we transmit the first part to the server for aggregation. After that, we pass the output of aggregated weights to the second part. This signals the conclusion of the forward pass, and now, the typical backward propagation takes place to fine-tune the customized layer.

Calculating the threshold and transmitting the data to the server before each round might increase the frequency of communication. Therefore, to do this, we adhere to a policy of “N” round transfer learning method, in which we first determine the threshold for each “N” round (if N equals 5, N equals 5, 10, 15, etc.) and then proceed to carry out transfer learning on the global model. To determine the threshold for the training rounds, we first determine the difference between the metrics of the current round and those of the prior round, and we sum the differences till the multiples of the “N” round are reached. Then, we compare this difference to the default threshold. Later, we set the difference as 0, and the same procedure repeats until the next “N” rounds.

**Algorithm 1:**
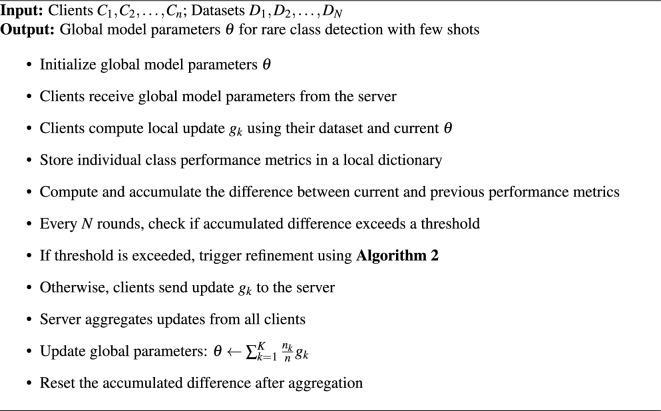
Overview of procedure for the proposed FL-based NIDS model.

**Algorithm 2:**
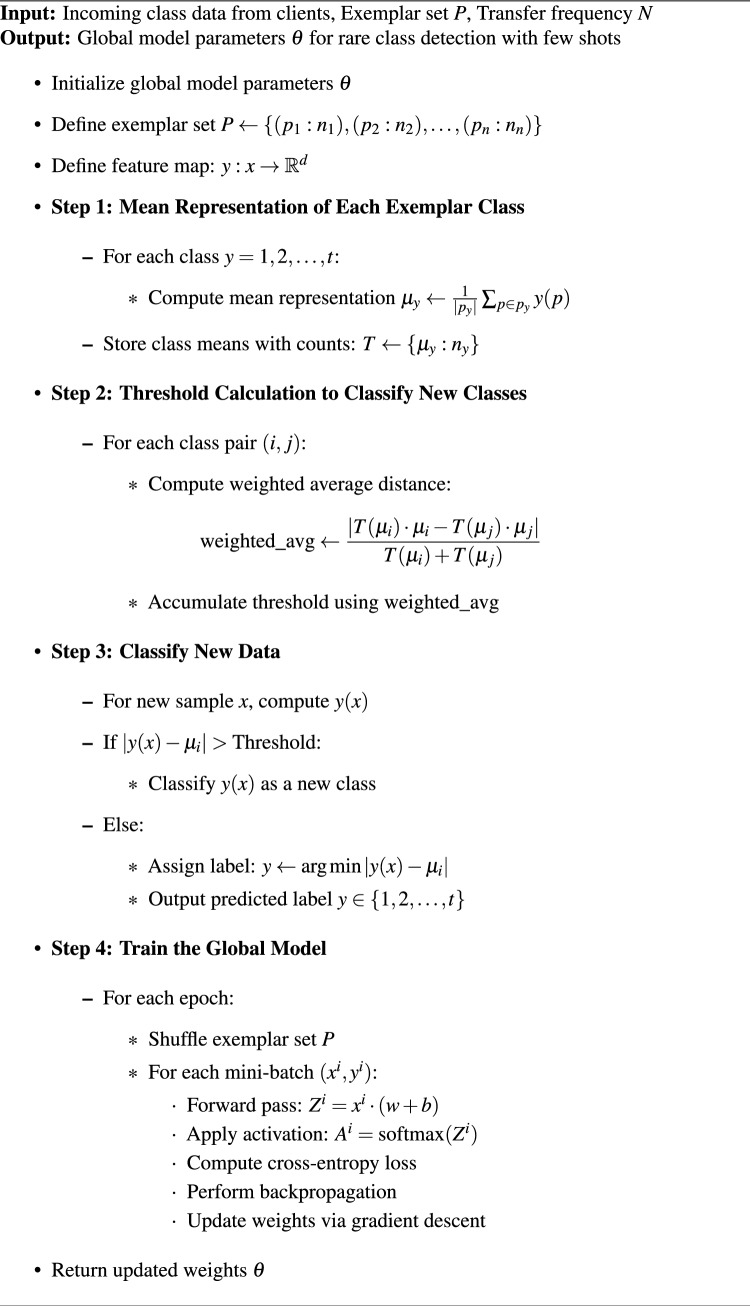
Server side procedure for the proposed FL-based NIDS model using personalized layers from TL.

### Global model

This algorithm 1 describes the procedure for training a FL-based NIDS tailored for detecting rare classes with few-shot learning. The process begins by initializing global model parameters, which are shared with multiple participating clients. In each round, each client computes an update based on its local dataset using the current global model. Clients then evaluate and store performance metrics for each class, focusing on the difference between the current and previous round’s performance. If the performance difference exceeds a predefined threshold after a set number of rounds, the algorithm repeats a specific procedure (Algorithm 2). Otherwise, clients send their updates to the server, where the global model parameters are aggregated based on weighted client updates. This iterative process continues until the global model, capable of accurately detecting rare intrusions with limited data, is sufficiently trained.

The algorithm 2 outlines the server-side procedure for the proposed FL-based NIDS model using personalized layers from Transfer Learning (TL). Initially, the global model parameters $\theta$ are initialized, and the exemplar set *P* is established, mapping each exemplar $p_i$ to its class $n_i$. A feature map $y: x \rightarrow \mathbb {R}^d$ is defined to transform incoming data *x* into the feature space $\mathbb {R}^d$. A few exemplars from each class are stored on the server before the training begins.

In Step 1, the mean representation $\mu _y$ of each exemplar class is computed by averaging the feature vectors of all exemplars in the class. A dictionary *T* stores these mean representations and their counts. The embedding representations per class are derived from the specified exemplars by inputting them into the global model, and the resulting output, without using the softmax function, serves as the embedding representation. In each class, a single representation is computed by taking the average of all the individual representations to prevent the occurrence of duplicate classes in the one hot encoding conversion when new classes are received from clients.

In Step 2, a threshold for classifying new data is calculated. For each class, the weighted average of differences between mean representations is computed, and the sum of these weighted averages determines the threshold. Before server initialization, the threshold value is acquired based on the embedding representations of each exemplar class. Consequently, the disparity between each example class is calculated, followed by the weighted average of these disparities, which yields the threshold value for the embedding. Over this threshold, data points are categorized as belonging to a new class.

In Step 3, new data is classified by comparing the distance of its feature representation from each class mean to the threshold: if the distance exceeds the threshold, the data is classified as a new class; otherwise, it is assigned to the nearest existing class based on the minimum distance. Upon receiving the data for the new class, the initial step is to acquire the embedding representation of the data and compare it with the current embedding representations. If the disparity between the embedding representations exceeds the predetermined threshold, the data is included as a new class.

Finally, Step 4 trains the global model over multiple epochs. During each epoch, the exemplar set *P* is shuffled. The algorithm performs forward propagation for each mini-batch to compute outputs using a softmax function, computes loss via cross-entropy, and updates model weights through backpropagation and gradient descent. There are no differences between the architecture of the client model and global models’ architecture, except for the individualized layers. A single instance of hot encoding is carried out at this stage, during which the new classes transmitted from the client are added to the server dataset as a new class.

### New class detection

We use clear-cut criteria based on performance metrics to determine the novelty or rarity of classes. When the model encounters deployment attack data on the client side, it is likely to classify a new class along with the existing classes falsely. Consequently, the ongoing attacks lead to a decline in the performance indicators of the current class. Therefore, in this section, we uphold a state dictionary that encompasses the performance metrics of various classes, such as accuracy, recall, and F1-score (explained in “Experimental methodology” section). Subsequently, following each iteration, we calculate the disparity between the metrics of the present iteration and those of the preceding iteration. We can infer the recognition of a novel class if the disparity exceeds the threshold value. Subsequently, the data from the recently identified class is encrypted and delivered to the server, where the global model is situated, as shown in Fig. 1. The training will continue normally if the disparity is less than the default threshold.

### Complexity analysis

We analyze the time complexity of each step in the algorithm to understand its overall computational cost.

- **Initialization:** Initializing the model parameters and exemplar set is a straightforward operation with constant time complexity, $O(1)$.
- **Mean Calculation for Each Class (Step 1):** We calculate the mean of all exemplars in each of the $t$ classes. If each class has $n_y$ exemplars, this operation takes $O(t \cdot n_y)$ time.
- **Threshold Calculation for New Class Detection (Step 2):** Calculating thresholds involves comparing pairs of class means. With $t$ classes, this pairwise comparison yields a complexity of $O(t^2)$.
- **Classification of New Data (Step 3):** Each new data point is compared to the mean of each class, resulting in $O(t)$ complexity per classification.
- **Model Training (Step 4):** Training the global model over $T$ epochs and $m$ mini-batches requires iterating through the exemplar set $P$ in each batch. This results in a complexity of $O(T \cdot m \cdot |P|)$, where $|P|$ represents the size of the exemplar set.

**Overall Complexity:** The most computationally intensive part is model training (**Step 4**) with complexity $O(T \cdot m \cdot |P|)$. Therefore, the overall complexity of the algorithm is:

$$O(t \cdot n_y + t^2 + T \cdot m \cdot |P|)$$

## Experimental methodology

### Dataset preparation

Data transformation is undertaken to normalize and standardize dataset attributes, reducing dimensionality and ensuring consistency across numerical properties. Specifically, a MinMax scaler is applied to rescale features to a normalized range of [0, 1], enhancing uniformity in attribute distribution. During data preprocessing, rows containing “NaN” values are removed to preserve dataset integrity. To enable multi-class classification, one-hot encoding is employed, producing categorical labels for each incident to accommodate the classification model’s requirements. Feature engineering techniques are then applied, extracting only the most relevant columns instead of fitting each feature individually, thus optimizing model complexity and improving classification performance over unprocessed data. Furthermore, a correlation analysis isolates attributes with significant relevance to intrusion detection, removing features below a pre-defined correlation threshold. This process yields a streamlined, high-quality feature set that enhances the model’s efficacy and computational efficiency.

For simplicity, we use custom DNNs for different datasets. One model is designed for the CSE-CICIDS 2018 dataset, while another is used for the UNSW-NB15 and Edge-IIoT datasets, which is a feedforward neural network structure specifically intended for multi-class classification. Table 4, shows the architecture of model 1 for the CICIDS 2018 dataset, with four highly interconnected layers. The first layer, serving as the input layer, contains 23 neurons corresponding to the input dimensions. The following layers have 20, 10, and 5 neurons, each employing the rectified linear unit (ReLU) activation function, bringing non-linearity to the model. The last layer, which we mostly use as a personalization layer, has neurons depending upon the number of classes the client has in the testing dataset and employs the softmax activation function to provide output probabilities for various classes. The model is trained via the sparse categorical cross-entropy loss function and optimized through the Adam optimizer.

**Table 4:** Model 1 architecture.

Type	Parameters
Dense (ReLU)	20 units, input_dim = 23
Dense (ReLU)	10 units
Dense (ReLU)	5 units
Dense (Softmax)	20 units

Table 5 shows the architecture of Model 2 used on UNSW NB-15 and Edge-IIot datasets, which includes several dense layers interspersed with batch normalization, LeakyReLU activation functions, and dropout layers to prevent overfitting. The model ends with a softmax layer with 20 units for multiclass classification.

**Table 5:** Model 2 architecture.

Type	Parameters
Dense	64 units, input_dim = 22
BatchNormalization	–
LeakyReLU	alpha = 0.1
Dropout	0.3
Dense	128 units
BatchNormalization	–
LeakyReLU	alpha = 0.1
Dropout	0.4
Dense	64 units
BatchNormalization	–
LeakyReLU	alpha = 0.1
Dropout	0.3
Dense (Softmax)	20 units

Before commencing the FL process, the server calculates the class threshold, while the client sets a fixed performance threshold and defines “N” to regulate the frequency of transfer learning. Following this, the training process begins. The experiments use a laptop equipped with a Ryzen 5 4600H CPU processor and 16GB of RAM. The tests are conducted locally on the CPU using the VSCODE editor.

Evaluation metrics are used to measure the effectiveness and performance of intrusion detection systems (IDSs) in detecting and preventing malicious attacks on computer networks. There are different ways of writing evaluation metrics for network intrusion detection systems, depending on the type of IDS, the data used, and the objectives of the evaluation. Here are some examples of evaluation metrics for network intrusion detection systems:

Accuracy: This metric measures the proportion of correctly classified network packets out of the total number of packets. It is calculated as:

2 $$\begin{aligned} Accuracy = \frac{TP + TN}{TP + TN + FP + FN} \end{aligned}$$

where TP: True Positives (correctly classified as an attack); TN: True Negatives (correctly classified as benign); FP: False Positives (wrongly classified as an attack); FN: False Negatives (wrongly classified as benign).

F-score: This metric combines the precision and recall of the IDS, which measure the accuracy and completeness of the detection, respectively. It is calculated as:

3 $$\begin{aligned} F-score = \frac{2PrecisionRecall}{Precision + Recall} \end{aligned}$$

where Precision is the proportion of correctly detected anomalous packets out of the total number of detected anomalous packets, and Recall is the proportion of correctly detected anomalous packets out of the total number of actual anomalous packets. Precision and Recall are calculated as follows:

4 $$\begin{aligned} Precision= & \frac{True Positives}{False Positives + True Positives} \end{aligned}$$

5 $$\begin{aligned} Recall= & \frac{True Positives}{False Negatives + True Positives} \end{aligned}$$

## Experimental results

### Proposed results

The experimental results on three datasets **CSE-CICIDS 2018**, **UNSW-NB15**, and **Edge-IIoT** illustrate the impact of integrating TL with FL in the proposed NIDS model. We set the frequency of TL as 3(N=3), which means after the completion of every 3rd round, the clients compute the difference between the performance metrics and check if the difference is more significant than the threshold. In this context, precision is primarily employed as the measure to calculate the difference, as it directly affects the effectiveness of the incorrectly categorized categories.

We have excluded specific attacks from some clients while including them in others to evaluate the model’s performance under conditions where some attack types are absent during training but appear in the test set (behave as unseen classes). This approach enables assessing the proposed model’s generalization and robustness in handling previously unseen attack types. Additionally, the client distribution across these datasets is heterogeneous, which helps demonstrate how the proposed federated learning model can effectively support clients with fewer samples, allowing them to perform well despite limited data.

This section examines the performance of both the naive federated model and the proposed enhanced model across three distinct datasets: **CSE-CICIDS 2018**, **UNSW-NB15**, and **Edge-IIoT**. By analyzing metrics like *precision*, *recall*, and *F1 score* for each class label, we explore why the proposed model performs consistently better and highlight the situations where the naive model falls short.

### Performance on CSE-CICIDS 2018 dataset

Table 6 depicts the results of results obtained on the 4 clients of the CSE-CICIDS 2018 dataset. In the dataset, the naive model’s performance was especially challenged by imbalanced data distributions across different attack types, such as **DDoS-HOIC** and **DoS-SlowHTTPTest**. For instance, the naive model yielded zero values for both precision and recall for the DDoS-HOIC attack, and for DoS-SlowHTTPTest, it only achieved a $50.41\%$ precision and $59.87\%$ recall. This poor performance stems from the naive model’s inability to adapt to data heterogeneity and its lack of specific handling mechanisms for minority classes, causing it to struggle with sparse data distributions and significantly lower F1 scores.

**Table 6:** Performance of each client in FL set-up using naive federated model and proposed model with each class label on CSE-CICIDS 2018 dataset.

Clients	Class labels	Naive federated model			Proposed model		
		Precision (%)	Recall (%)	F1 score (%)	Precision (%)	Recall (%)	F1 score (%)
Client1	Benign	93.27	90.01	91.62	98.24	95.87	97.04
	Bot	61.20	55.32	58.14	94.28	95.76	95.01
	DDoS-HOIC	0	0	0	97.01	99.18	98.08
	DDoS-LOIC HTTP	93.95	95.21	94.87	97.84	98.37	98.01
	DoS-HULK	98.40	99.89	99.03	99.03	100.00	100.00
	DoS-SlowHTTPTest	50.41	59.87	54.72	97.97	99.43	99.09
Client2	Benign	91.47	88.21	89.34	97.86	98.81	99.50
	Bot	64.05	56.51	60.12	89.01	96.51	92.36
	DDoS-HOIC	96.02	92.58	94.24	96.89	97.20	97.04
	DDoS-LOIC HTTP	0	0	0	99	99.70	100
	DoS-HULK	98.68	99.92	99.42	100	100	100
	DoS-SlowHTTPTest	59.63	56.23	57.89	99.10	99.0	99.11
Client3	Benign	90.78	93.92	92.35	96.42	97.87	97.14
	Bot	60.98	68.43	64.57	89.01	87.47	88.23
	DDoS-HOIC	93.67	96.59	95.12	96.34	99.04	97.67
	DDoS-LOIC HTTP	91.22	94.53	92.84	95.63	93.95	94.78
	DoS-HULK	0	0	0	97.89	99.16	98.52
	DoS-SlowHTTPTest	55.78	51.01	53.14	73.41	75.24	74.32
Client4	Benign	90.21	93.49	91.84	97.55	96.02	96.78
	Bot	59.83	56.11	57.91	89.11	86.18	87.62
	DDoS-HOIC	96.24	92.65	94.39	96.32	95.59	95.95
	DDoS-LOIC HTTP	93.85	96.21	95.01	93.67	95.16	94.41
	DoS-HULK	98.91	100	99.54	99.13	100	99.82
	DoS-SlowHTTPTest	0	0	0	98.01	97.28	97.64

In contrast, the proposed model demonstrated considerable improvements across all metrics. For the DDoS-HOIC attack, it achieved precision and recall values of **97.01%** and **99.18%**, respectively, leading to an F1 score of **98.08%**. Similarly, for DoS-SlowHTTPTest, the proposed model reached a precision of **97.97%** and recall of **99.43%**, resulting in an F1 score of **99.09%**—an increase of over $40\%$ in F1 scores compared to the naive model. These enhancements are primarily due to the proposed model’s personalized adaptive parameter-sharing strategies, which enable it to retain knowledge from minority classes and adapt to data variability across clients.

**Fig. 2:**
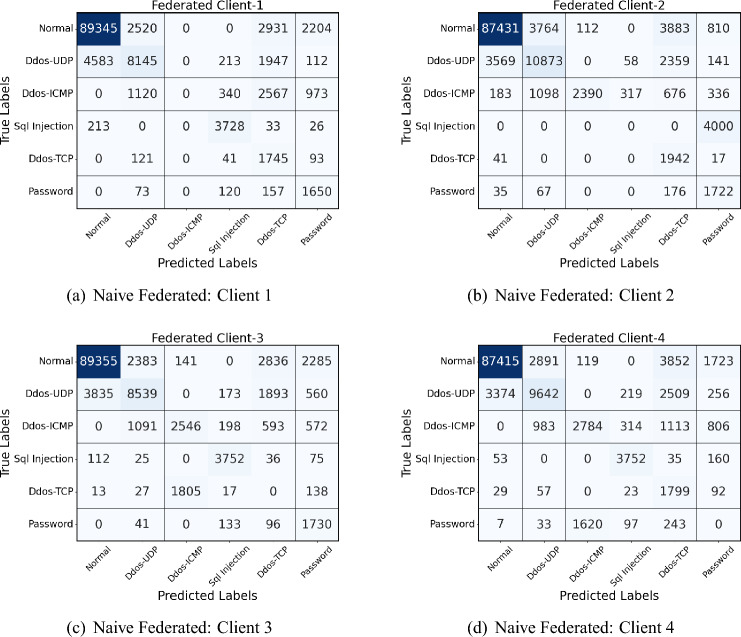
Confusion matrices of all 4 clients in Naive federated model of CSE-CICIDS-2018 dataset.

**Fig. 3:**
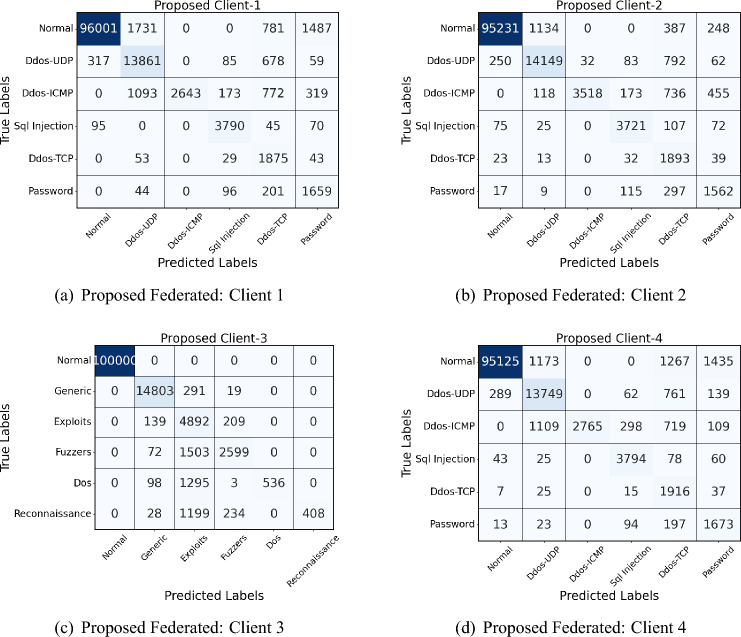
Confusion matrices of all 4 clients in proposed model of CSE-CICIDS-2018 dataset.

In the confusion matrix analysis of the naive Federated model from Fig. 2 and Proposed models from Fig. 3 across various client configurations, confusion matrices reveal key performance distinctions, particularly in intrusion class identification accuracy. The “Benign” class consistently displays high detection rates, with the Proposed model achieving further improvements due to minimized misclassification across clients. Notably, specific attack types, such as “DDoS-HOIC” and “DDoS-LOIC HTTP,” are accurately captured in the Proposed model, with lower false negatives and improved row consistency in confusion matrices across all clients. These advancements reflect the effectiveness of the personalized model in maintaining accurate classification, even for more complex or less frequent attacks like “DoS-SlowHTTPTest”.

The improved detection accuracy for classes initially absent or misclassified in Federated settings-such as zero detections in Federated for specific attacks, which the Proposed model effectively captures-underlines the utility of new class method detection detailed in algorithm 2. For instance, “DoS-SlowHTTPTest,” missing in Federated Client-4, is captured in the Proposed model with high accuracy. These results highlight the Proposed model’s robustness in accurately identifying diverse attacks, thus enhancing the system’s adaptive security capabilities for heterogeneous intrusion scenarios.

### Performance on UNSW-NB15 dataset

Table 7 depicts the results obtained on the four clients of the UNSW NB-15 dataset. The UNSW-NB15 dataset posed significant challenges for the naive model, particularly with infrequent attack types like **Exploits**, **Fuzzers**, and **DoS**. For example, the naive model achieved zero values for both precision and recall on Fuzzers and DoS classes, leading to zero F1 scores. For Exploits, the naive model yielded a precision of $59.31\%$ and a recall of $60.02\%$, resulting in a modest F1 score of $59.11\%$. These results highlight the naive model’s difficulty in learning from sparse and imbalanced data distributions, as it lacks mechanisms to balance parameter sharing when class frequencies are uneven across clients.

**Table 7:** Performance of each client in FL set-up using naive Federated model and proposed model with each class label on UNSW-NB15 dataset.

Clients	Class labels	Naive federated model			Proposed model		
		Precision (%)	Recall (%)	F1 score (%)	Precision (%)	Recall (%)	F1 score (%)
Client1	Normal	100	100	100	100.00	100.00	100.00
	Generic	91.34	98.00	95.12	95.78	91.53	94.76
	Exploits	0.00	0.00	0.00	82.32	88.67	85.14
	Fuzzers	78.42	72.29	75.51	79.38	79.51	76.84
	Dos	28.76	68.10	39.45	37.13	72.88	41.95
	Reconnaissance	42.51	68.24	52.12	49.76	63.58	55.04
Client2	Normal	100	100	100	100.00	100.00	100.00
	Generic	97.05	98.45	97.62	92.47	96.12	94.03
	Exploits	59.31	60.02	59.11	65.18	57.96	60.78
	Fuzzers	0.00	0.00	0.00	87.56	89.22	89.10
	Dos	37.21	38.42	37.11	40.73	34.89	39.04
	Reconnaissance	27.88	79.45	41.32	29.91	81.55	47.38
Client3	Normal	100	100	100	100.00	100.00	100.00
	Generic	96.54	98.07	98.14	93.27	92.88	94.58
	Exploits	46.93	92.77	61.21	50.92	88.56	69.12
	Fuzzers	82.61	57.48	68.89	87.97	61.25	73.09
	Dos	0.00	0.00	0.00	87.13	79.45	82.76
	Reconnaissance	0.00	0.00	0.00	78.33	74.92	76.19
Client4	Normal	100	100	100	100.00	100.00	100.00
	Generic	98.76	97.01	98.34	95.55	94.37	96.42
	Exploits	57.08	66.74	62.01	60.77	63.58	62.78
	Fuzzers	77.89	65.67	71.22	73.44	74.91	72.56
	Dos	35.92	72.45	47.84	39.55	69.21	53.90
	Reconnaissance	0.00	0.00	0.00	81.89	78.57	80.98

In comparison, the proposed model showed marked improvements in these challenging classes. For the Fuzzers attack type, it achieved precision and recall scores of **87.56%** and **89.22%**, respectively, with an F1 score of **89.10%**—an increase of nearly $50\%$ over the naive model. Similarly, for Exploits, the proposed model reached a precision of **82.32%** and a recall of **88.67%**, resulting in an F1 score of **85.14%**. These improvements stem from the proposed model’s incremental FL approach, which enables more effective handling of uncommon attack types by learning and retaining representations through iterative parameter-sharing. The proposed model achieves consistent recall and precision rates by preserving learned information specific to rare attack patterns, even for minority classes.

**Fig. 4:**
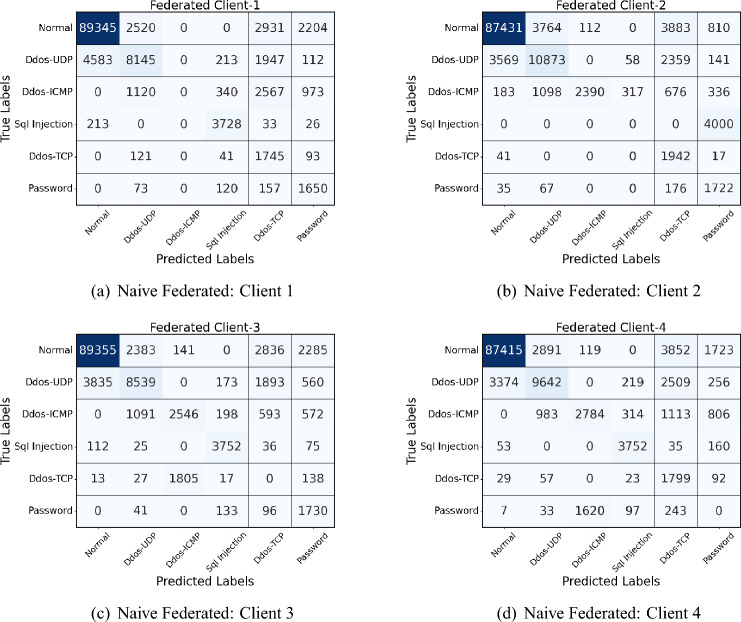
Confusion matrices of all 4 clients in Naive federated model of UNSW NB-15 dataset.

**Fig. 5:**
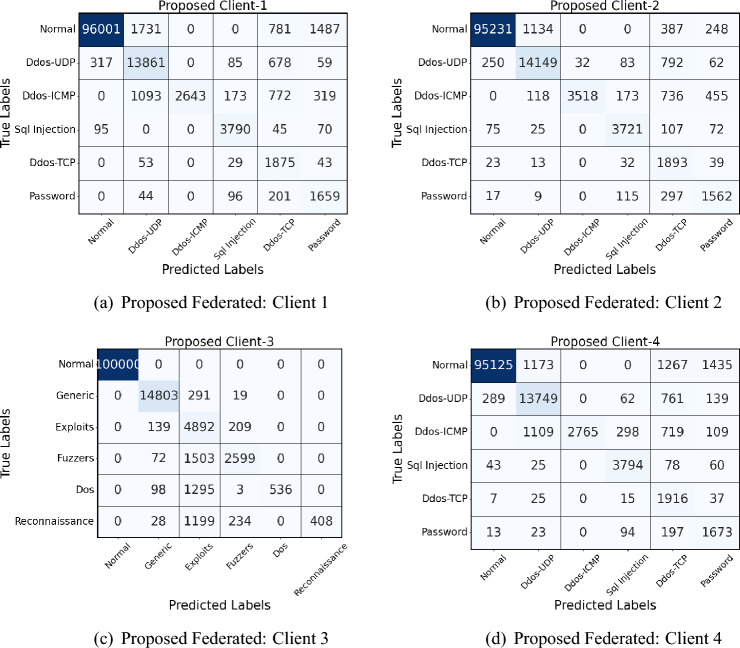
Confusion matrices of all 4 clients in proposed model of UNSW NB-15 dataset.

Figures 4 and 5 show the confusion matrices of naive and proposed federated models conducted on the UNSW NB-15 dataset. As depicted in the figures, overall, the proposed model improves classification accuracy, particularly in reducing misclassifications across non-normal classes. For instance, in Client-1, the proposed model decreases misclassification in the “Exploits” and “Dos” classes, indicated by fewer off-diagonal entries in these categories compared to the naive approach. Similarly, for Client-2, the proposed model shows improved identification of “Generic” and “Reconnaissance” classes, with lower counts of misclassified samples in those rows. Client-3 exhibits a reduction in misclassified “Generic” samples, and Client-4 shows fewer errors in the “Exploits” class with the proposed model. The performance gains are most evident in off-diagonal reductions, showing that the proposed model achieves better specificity and accuracy across the diverse categories. The results highlight the proposed model’s enhanced ability to capture nuanced patterns across different attack types, making it more reliable in a federated learning setting with diverse clients.

### Performance on Edge-IIoT dataset

Table 8 shows the results obtained on the four clients of the Edge IIoT dataset. The Edge-IIoT dataset further underscores the naive model’s limitations, particularly with complex attack patterns like **DDoS-ICMP** and **SQL Injection**. For example, the naive model reported zero values for both precision and recall on the DDoS-ICMP attack, mainly due to its standard parameter-sharing method that does not prioritize the retention of information about rare patterns across clients. Similarly, in the case of SQL Injection, the naive model achieved only a $51.32\%$ precision and $60.71\%$ recall, resulting in a suboptimal F1 score of $55.65\%$.

**Table 8:** Performance of each client in FL set-up using naive federated model and proposed model with each class label on Edge-IIot dataset.

Clients	Class labels	Naive Federated model			Proposed model		
		Precision (%)	Recall (%)	F1 score (%)	Precision (%)	Recall (%)	F1 score (%)
Client1	Normal	92.34	89.12	90.70	97.58	96.01	96.79
	DDos-UDP	63.19	54.45	58.46	90.52	91.81	91.16
	DDos-ICMP	0	0	0	54.31	52.78	53.53
	SQL Injection	92.85	93.10	92.97	95.84	96.12	95.98
	DDos-TCP	98.14	98.94	98.54	99.04	100.00	99.52
	Password	51.32	60.71	55.65	86.78	88.91	87.83
Client2	Normal	90.47	87.31	88.86	96.74	97.63	97.18
	DDos-UDP	64.92	57.23	60.85	88.75	95.37	91.94
	DDos-ICMP	94.88	90.56	92.67	96.78	96.45	96.61
	SQL Injection	0	0	0	58.65	59.32	58.98
	DDos-TCP	97.84	98.65	98.24	99.13	99.54	99.34
	Password	57.84	55.18	56.47	93.76	92.42	93.09
Client3	Normal	89.56	92.34	90.92	95.78	97.12	96.44
	DDos-UDP	61.52	67.18	64.21	88.19	86.98	87.58
	DDos-ICMP	91.82	94.35	93.07	95.56	97.23	96.39
	SQL Injection	90.45	92.72	91.57	93.84	94.10	93.97
	DDos-TCP	0	0	0	59.78	58.94	59.36
	Password	54.29	50.21	52.17	74.36	76.49	75.41
Client4	Normal	89.71	92.67	91.16	96.89	95.23	96.06
	DDos-UDP	58.92	55.34	57.08	88.31	85.74	87.00
	DDos-ICMP	94.67	91.83	93.23	95.42	94.82	95.12
	SQL Injection	93.18	95.42	94.28	93.10	94.32	93.71
	DDos-TCP	98.41	99.56	98.98	98.88	99.43	99.15
	Password	0	0	0	69.45	70.12	69.78

The proposed model, however, excelled in handling these complex and sparse attacks due to its fine-grained parameter-sharing strategy. For DDoS-ICMP, it achieved precision and recall scores of **54.31%** and **52.78%**, respectively, and for SQL Injection, it reached precision and recall scores of **95.84%** and **96.12%**, leading to an F1 score of **95.98%**. These values represent a significant improvement over the naive model, which achieved zero scores in some cases and lower than $60\%$ in others. The improvements of the proposed model can be attributed to its effective handling of rare patterns through custom parameter sharing, allowing it to retain specific features relevant to infrequent classes between clients. This results in more balanced learning outcomes, mainly when dealing with complex data in federated environments.

**Fig. 6:**
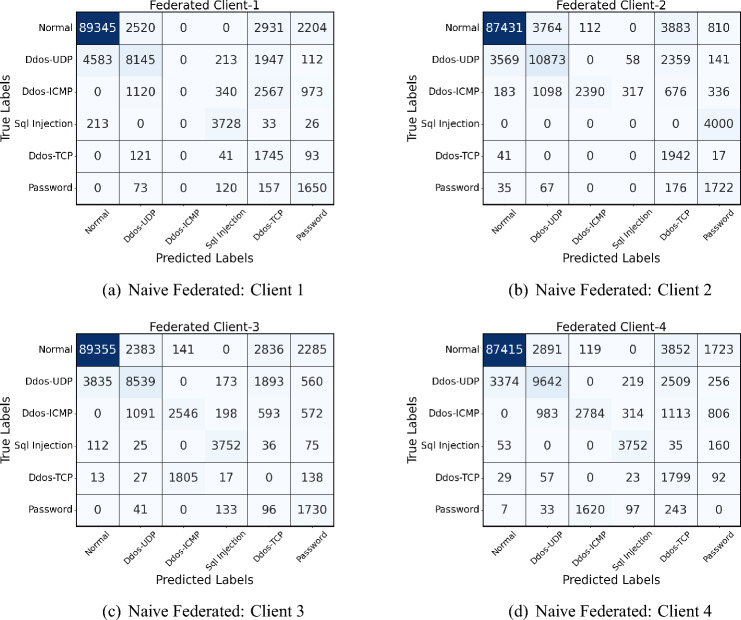
Confusion matrices of all 4 clients in Naive federated model of Edge-IIoT dataset.

**Fig. 7:**
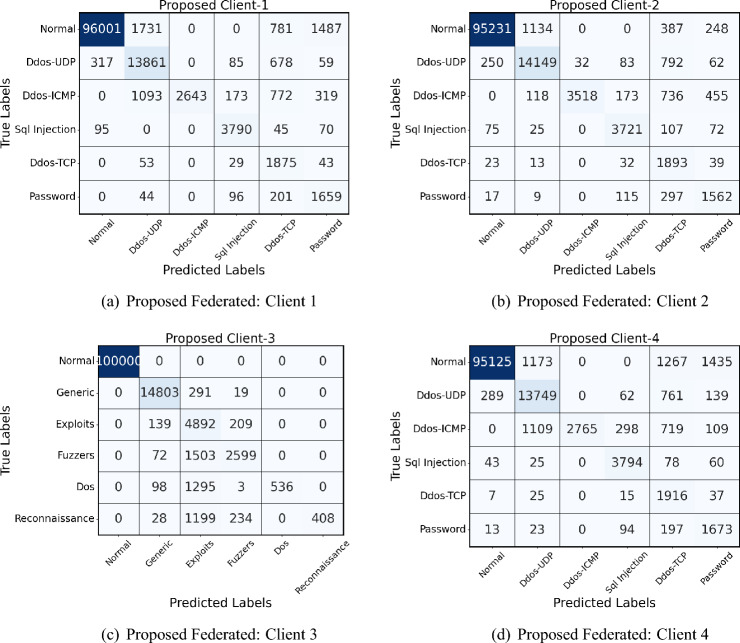
Confusion matrices of all 4 clients in proposed model of Edge-IIoT dataset.

The confusion matrix analysis of the proposed and federated (personalized) models in four clients in Fig. 6 (naive federated model) and Fig. 7 (proposed model) reveals notable improvements in network intrusion detection, especially for classes previously difficult to detect. In the personalized model, detection accuracy for the “Normal” traffic class is consistently high, with improvements seen across all clients; for example, Client-1’s correctly classified instances of Normal traffic increase from 89,345 in the Federated model to 96,001 in the proposed model. A similar pattern appears for DDoS-UDP and DDoS-TCP attacks, where the proposed model reduces misclassification rates. The personalized model demonstrates an improved detection rate for attack classes such as SQL Injection, which initially had zero true positives in several Federated matrices. This enhancement is due to a novel class detection method integrated into the proposed model, enabling the identification of previously undetected attack types.

### Summary and comparative analysis

The analysis of the performance of the proposed model versus the naive federated model across three datasets-CSE-CICIDS 2018, UNSW-NB15, and Edge-IIoT-reveals the critical role of an integrated new-class detection and adaptive learning mechanism in federated environments. The naive federated model’s inability to detect or learn from novel classes encountered during testing is mainly due to its static parameter-sharing mechanism, which assumes that client datasets have overlapping and consistent classes. Consequently, in cases where attack classes are missing from a client’s training data, the naive model fails to identify these classes in testing, leading to poor precision, recall, and F1 scores for infrequent or unseen classes like DDoS-HOIC in CSE-CICIDS and SQL Injection in Edge-IIoT.

The proposed model overcomes these limitations through a novel class detection algorithm, which triggers a review of newly emerging patterns or classes after every N federated round. If a new class is detected, the proposed model initiates a lightweight transfer learning phase, selectively transferring pertinent knowledge from other clients where this class is present, enabling rapid adaptation without full retraining. This approach allows the model to dynamically update its knowledge base, even for classes previously unseen by specific clients. This improves performance metrics, especially in minority classes that traditional models often overlook. For instance, in the UNSW-NB15 dataset, the naive model struggled with classes such as Exploits and Reconnaissance, frequently producing zero scores. In contrast, the proposed model maintained high precision and recall by successfully adapting to these classes as they were identified.

The proposed model’s incremental learning mechanism also reinforces the retention of knowledge on both common and rare attacks, which is further enhanced by selective parameter aggregation. This process ensures that critical parameters related to emerging classes are neither diluted nor lost over multiple federated rounds. For instance, the Edge-IIoT dataset, classes like Password attacks and DDos-ICMP, often underrepresented, were accurately detected and learned by the proposed model, which achieved significantly higher F1 scores by continuously accumulating and selectively aggregating class-specific parameters from various clients. This parameter retention effectively mitigates the common problem in naive models, where rare or sparse classes are either ignored or misclassified due to the limitations of static model aggregation.

Additionally, the proposed model’s adaptive parameter-sharing mechanism, paired with new-class detection and selective transfer learning, enhances learning in heterogeneous data conditions commonly seen in federated networks. This is evident in the CSE-CICIDS dataset, where the proposed model achieved near-perfect recall and F1 scores for challenging classes like DoS-SlowHTTPTest and Bot, as it continuously retained and reinforced knowledge from clients with varied data distributions. This ability to dynamically adapt, detect new courses, and reinforce sparse data patterns underlines the proposed model’s improved efficacy in federated environments, where heterogeneous data and novel class emergence are frequent. This adaptive and proactive approach equips the model with enhanced resilience against class imbalance and data sparsity challenges, offering a robust solution for real-world applications requiring high security, adaptability, and precision.

### Ablation study

To test the effectiveness of our proposed model, we perform an ablation study on the existing models such as FedGAN-IDS^41^, FGAN^42^ and IotDefender^20^ along with the centralized and the Base Federated Averaging(FedAvg)^7^. Our ablation study effectively compares the performance of your proposed model against existing models in the context of the CSE-CICIDS 2018 dataset.

To assess the effectiveness of our proposed model, we conducted an ablation study against several existing models: FedGAN-IDS^41^, FGAN^42^, IoTDefender^20,31^ and the centralized and Base Federated Averaging (FedAvg)^7^ approaches. The results of this study for two client cases are illustrated in Table 9.

1. Centralized Model Performance: The centralized model initially achieved a high accuracy of 97.57%. However, its performance metrics, particularly the F-1 Score (82.25%), Precision (82.14%), and Recall (81.91%), indicate a significant limitation in classifying unseen classes. This performance drop highlights the centralized model’s inability to generalize well when faced with novel data instances, a critical drawback in real-world scenarios where new attack types may emerge.
2. Federated Learning Performance: The FedAvg model exhibited an accuracy of 98.57%, but it struggled with lower F-1 Score (68.50%) and Precision (66.84%), suggesting that while it was able to leverage federated learning benefits, it did not adequately capture the underlying patterns in the data. The Recall of 67.05% further confirms that the model missed many relevant instances, emphasizing the challenges of balancing model performance in a federated setting.
3. Comparison with Existing Models: FedGAN-IDS and FGAN showed lower performance metrics, with FedGAN-IDS achieving 91.57% accuracy and an F-1 Score of 79.50%. FGAN performed slightly worse, with an accuracy of 90.21% and an F-1 Score of 72.51%. These results indicate that while both models provide valuable frameworks for intrusion detection, they struggle to match the robustness of our proposed model, particularly in handling diverse attack classes. IoTDefender and FL-TL CNN showed relatively better performance with an accuracy of 95.40% and an F-1 Score of 80.24%, but it still falls short compared to our proposed model.
4. Proposed Model Superiority: Our proposed model outperformed all the existing models, achieving the highest accuracy of 98.90%, an impressive F-1 Score of 95.79%, Precision of 94.51%, and Recall of 93.01%. This significant enhancement in performance metrics can be attributed to our model’s design, which effectively captures complex relationships within the data, allowing it to generalize better to unseen classes.

**Table 9:** Performance comparison of proposed model with existing models on CSE-CICIDS 2018 dataset.

Model	Accuracy (%)	F-1 score (%)	Precision (%)	Recall (%)
Centralized	97.57	82.25	82.14	81.91
FedAvg^7^	98.57	68.50	66.84	67.05
FedGAN-IDS^41^	91.57	79.50	78.84	78.30
FGAN^42^	90.21	72.51	73.38	69.68
IoTDefender^20^	95.40	80.24	79.62	80.10
FL-TL-CNN^31^	96.12	89.41	87.49	84.29
Proposed model	**98.90**	**95.79**	**94.51**	**93.01**

Significant values are in bold.

The results of the ablation study provide compelling evidence of our proposed model’s superiority to existing approaches. The improved metrics not only demonstrate its effectiveness in recognizing known and unseen attack classes but also highlight its potential for real-world applications in network intrusion detection systems.

Ablation Study on UNSW-NB15 Dataset

To further validate the effectiveness of our proposed model, we conducted an ablation study on the UNSW-NB15 dataset, comparing it with existing models: Centralized, FedAvg^7^, FedGAN-IDS^41^, FGAN^42^, IoTDefender^20^, and FL-TL-CNN^31^. The performance metrics for this comparison are presented in Table 10.

1. Centralized Model Performance: The centralized model achieved an accuracy of 96.30%, along with an F-1 Score of 84.51%, Precision of 87.58%, and Recall of 81.27%. While these metrics are commendable, the centralized model still faces limitations in classifying unseen attack types. This may hinder its real-world applicability, especially in dynamic environments where new threats can emerge.
2. Federated Learning Performance: The FedAvg model demonstrated a lower accuracy of 93.50% and an F-1 Score of 80.20%. The Precision (83.00%) and Recall (77.00%) metrics further illustrate that the FedAvg model struggles to effectively capture the nuances of the data, which can lead to missed detections in critical scenarios.
3. Comparison with Existing Models: FedGAN-IDS and FGAN yielded better results than the centralized and FedAvg models. FedGAN-IDS achieved an accuracy of 97.57% and an impressive F-1 Score of 90.50%, indicating a robust performance in identifying various attack classes. Similarly, FGAN recorded an accuracy of 96.57% with an F-1 Score of 92.50%, demonstrating its effectiveness in intrusion detection tasks. IoTDefender matched FedGAN-IDS with an accuracy of 97.57% and an F-1 Score of 92.51%, showcasing its potential for real-time intrusion detection in IoT environments. However, while these models perform well, they still do not surpass the capabilities of our proposed—FL-TL-CNN model across key metrics, demonstrating improvements across all evaluated aspects. In terms of accuracy, the Proposed Model achieved 98.70, a noticeable increase over FL-TL-CNN’s 97.19. Precision rose from 90.05 in FL-TL-CNN to 95.41 in the Proposed Model, marking a 5.36% improvement. Similarly, recall saw a substantial rise from 91.41 to 94.34, while the F1 score increased from 92.02 to 96.03, indicating our model’s enhanced effectiveness in correctly classifying instances and reducing misclassification.
4. Proposed Model Superiority: Our proposed model outperforms all existing models with an accuracy of 98.70%, a remarkable F-1 Score of 95.41%, a Precision of 94.34%, and a Recall of 96.03%. This significant enhancement in performance metrics can be attributed to our model’s architecture, which effectively leverages advanced learning techniques to adapt to the complexities of the data and improve generalization to unseen attack classes.

**Table 10:** Performance comparison of proposed model with existing models on UNSW-NB15 dataset.

Model	Accuracy (%)	F-1 Score (%)	Precision (%)	Recall (%)
Centralized	96.30	84.51	87.58	81.27
FedAvg^7^	93.50	80.20	83.00	77.00
FedGAN-IDS^41^	97.57	90.50	92.84	88.35
FGAN^42^	96.57	92.50	91.84	93.37
IoTDefender^20^	97.57	92.51	91.58	93.24
FL-TL-CNN^31^	97.19	90.05	91.41	92.02
Proposed model	**98.70**	**95.41**	**94.34**	**96.03**

Significant values are in bold.

The results of the ablation study on the UNSW-NB15 dataset reinforce the effectiveness of our proposed model compared to traditional and contemporary models. The superior performance metrics not only highlight its capacity for accurate intrusion detection, but also demonstrate its readiness for deployment in real-world applications, where adaptability to new and evolving threats is crucial. Performance metrics for this comparison on the Edge IIoT dataset are presented in Table 11.

1. Centralized Model Performance: The centralized model achieved an accuracy of 95.13%, with an F-1 Score of 82.87%, Precision of 86.04%, and Recall of 80.01%. Although the centralized approach provides solid performance, its lower recall indicates limitations in identifying all attack types, particularly rare or unseen attacks. This restricts the model’s utility in dynamic environments where real-time detection of new attack types is crucial.
2. Federated Learning Performance (FedAvg): The FedAvg model, configured with six clients, reported an accuracy of 91.95%, with an F-1 Score of 78.33%, Precision of 80.84%, and Recall of 76.08%. These metrics reflect a moderate detection capability but highlight challenges adapting to complex data nuances. The lower recall and F-1 score suggest that FedAvg may struggle to capture critical attack patterns, potentially leading to missed detections in sensitive, high-stakes scenarios.
3. Comparison with Existing Models: FedGAN-IDS: FedGAN-IDS demonstrated notable improvements with an accuracy of 96.47% and an F-1 Score of 88.76%. The higher precision (91.08%) and recall (87.03%) underscore its effectiveness in accurately identifying various attack classes. However, while it excels in general performance, some rare attack classes may still pose a challenge. FGAN: The FGAN model achieved an accuracy of 95.61% and an F-1 Score of 90.28%, with a high recall of 91.33%. This performance indicates that FGAN is particularly adept at handling rare classes, achieving a balanced trade-off between precision and recall, which enhances its applicability for real-time intrusion detection. IoTDefender: IoTDefender matched FedGAN-IDS in accuracy, achieving 96.52% with an F-1 Score of 91.24%, Precision of 90.51%, and Recall of 92.01%. This demonstrates its strong detection capacity in IoT environments, particularly for real-time applications. However, despite its high performance, it falls short of the overall capabilities of the proposed model. The existing FL-TL-CNN model, across key evaluation metrics, demonstrates notable improvements. The Proposed Model achieved an accuracy of 97.92, a 2% increase over FL-TL-CNN’s 95.89. Precision also saw a significant gain, with the Proposed Model reaching 94.56 compared to FL-TL-CNN’s 90.35, representing an enhancement of 4.21%. In terms of recall, our model scored 93.41, which is 4.92% higher than FL-TL-CNN’s 88.49. Finally, the F1 score for the Proposed Model was 95.07, surpassing FL-TL-CNN’s 91.9 by 3.17%. These improvements underscore the enhanced capability of our model in effectively handling classification tasks on non-iid data.
4. Proposed Model Superiority: Our proposed model outperformed all other models with an accuracy of 97.92%, an F-1 Score of 94.56%, a Precision of 93.41%, and a Recall of 95.07%. This significant improvement can be attributed to our model’s architecture, which incorporates advanced learning techniques such as GAN-based oversampling and ensemble learning to effectively capture complex data patterns and adapt to unseen attack classes. The superior metrics underscore the proposed model’s robustness, adaptability, and reliability for deployment in dynamic, real-world environments where new threats may continuously emerge. The ablation study results on the Edge-IIoT dataset further validate the effectiveness of the proposed model in comparison to traditional and contemporary approaches. The model’s superior accuracy, recall, and F-1 score reflect its high capability in accurate and comprehensive intrusion detection and highlight its readiness for real-world deployment, where adaptability to emerging threats is essential.

**Table 11:** Performance comparison of proposed model with existing models on Edge-IIoT dataset.

Model	Accuracy (%)	F-1 score (%)	Precision (%)	Recall (%)
Centralized	95.13	82.87	86.04	80.01
FedAvg^7^	91.95	78.33	80.84	76.08
FedGAN-IDS^41^	96.47	88.76	91.08	87.03
FGAN^42^	95.61	90.28	89.97	91.33
IoTDefender^20^	96.52	91.24	90.51	92.01
FL-TL-CNN^31^	95.89	90.35	88.49	91.9
Proposed model	**97.92**	**94.56**	**93.41**	**95.07**

Significant values are in bold.

### Communication overhead and scalability in real-world settings

Although we did not conduct explicit scalability experiments, the communication efficiency of the proposed FL-TL model can be theoretically deduced. By employing a personalized federated averaging approach, only the parameters of the shared layers are transmitted between clients and the server, significantly reducing communication costs. Unlike conventional FL frameworks that require the transmission of the entire model, our method selectively shares only essential parameters, optimizing bandwidth utilization.

This selective transmission strategy provides two key advantages:

- *Reduced bandwidth consumption:* By minimizing the transmitted data volume, the proposed approach significantly lowers bandwidth requirements, enhancing its feasibility for deployment across large-scale distributed NIDS frameworks.
- *Improved resource utilization:* The model architecture consists of shared and personalized layers, allowing clients to train locally while transmitting only the shared layer updates to the server.

This separation of computational processes further contributes to:

- *Localized processing efficiency:* Clients can allocate computational resources to refining personalized layers, enabling effective learning from local data while avoiding redundant calculations.
- *Optimized server aggregation:* Since only the shared layer updates are transmitted, the computational burden on the server is reduced, facilitating faster global model convergence while maintaining model performance across heterogeneous network environments.

Scalability is critical in FL, especially with heterogeneous data distributions and many clients. Our model incorporates an “N-round transfer learning” strategy, which minimizes communication frequency by ensuring only significant updates are transmitted to the global model. Additionally, leveraging adaptive personalized federated averaging, the model effectively manages client heterogeneity and scales efficiently. This approach benefits network intrusion detection systems, where data sources vary across clients. The selective parameter-sharing strategy further reduces communication bottlenecks, making it viable for large-scale decentralized deployments. The framework ensures adaptability across diverse network environments by dynamically adjusting model aggregation frequency and optimizing communication overhead. This enables effective learning across various attack scenarios while maintaining computational efficiency, making it well-suited for real-world cybersecurity applications.

## Conclusion

This research introduces an advanced FL framework tailored for NIDS that significantly enhances the detection accuracy of rare and zero-day attack classes. Traditional machine learning approaches struggle with the limited and non-IID distribution of rare classes within large datasets, often resulting in performance degradation and a higher rate of false alarms. To address these challenges, we transitioned from conventional machine learning models to a federated architecture that aggregates knowledge from multiple clients while preserving privacy. This approach allows each client to retain its unique data patterns while the global model benefits from generalizable insights from the federation.

Key to this model’s success is the integration of personalized layers at the client level, which adaptively capture local data characteristics and infrequent attack patterns. Additionally, we implemented an “N” round transfer learning strategy to optimize communication and enhance scalability, transmitting only significant updates to the global model. This enables the model to effectively learn and transfer knowledge about rare and previously unseen classes, ensuring each client benefits from an enriched understanding of less prevalent attacks.

Our experimental evaluation on large-scale NIDS datasets, including CSE-CICIDS-2018, Edge IIoT, and UNSW NB-15, demonstrates the proposed model’s ability to detect rare and zero-day attack classes accurately asses. Results show substantial improvement over existing FL-based NIDS methodologies, with reduced false alarm rates and enhanced model adaptability. Furthermore, the customizable layers facilitate the model’s acquisition of new attack information without requiring prior examples during the testing. This allows it to identify novel attacks accurately once sufficient federation cycles are complete.

## Future work

This study bridges the essential gap in rare class detection within federated learning environments and sets the stage for further advancements in privacy-preserving, high-performance NIDS. By effectively addressing the challenges posed by dynamic data distributions and infrequent attack classes, this framework holds great potential for practical deployment across a wide range of real-world network security scenarios, providing a reliable and adaptive defense against emerging threats in diverse and evolving network environments.

Future work can focus on personalized federated learning, leveraging embedding representations for aggregation to reduce communication overhead and accelerate the process. Enhancing privacy in split learning can involve personalized federated aggregation through advanced encryption or differential privacy methods for safeguarding sensitive data. Additionally, dynamically adapting intrusion detection models to evolving attack patterns and network conditions may benefit from integrating reinforcement learning for autonomous parameter adjustments.

## Supplementary Information

Supplementary Information.

## Data Availability

The dataset analyzed during the current study is available at https://www.unb.ca/cic/datasets/ids-2018.html, https://research.unsw.edu.au/projects/unsw-nb15-dataset, https://www.kaggle.com/datasets/mohamedamineferrag/edgeiiotset-cyber-security-dataset-of-iot-iiot.

## Code availibility

https://github.com/Abhijit4-debug/FED-TL/tree/main.
