# Supplementary material for: Federated transfer learning for rare attack class detection in network intrusion detection systems
Source: Sci Rep. 2025 Sep 30;15:33797. doi: 10.1038/s41598-025-02068-x (PMC12484838; doi:10.1038/s41598-025-02068-x)
Supplement: Supplementary file 1 — Supplementary Information. [file 41598_2025_2068_MOESM1_ESM.pdf]

Federated Transfer Learning for Rare Attack Class Detection in Network Intrusion Detection Systems

Chunduru Sri Abhijit[1], Y Annie Jerusha[1], *S P Syed Ibrahim[1], and Vijay Varadharajan[2]

[1]School of Computer Science and Engineering, Vellore Institute of Technology, Chennai Campus, Chennai-600127, India
[2]Advanced Cyber Security Engineering Research Centre, The University of Newcastle, Callaghan, Australia
*Corresponding author: S P Syed Ibrahim, e-mail: syedibrahim.sp@vit.ac.in

Appendix

a Centralized Learning

Let's represent it with mathematical notations:

Let $D = \{D_1, D_2, \dots, D_N\}$ represent network traffic data collected from N network points. Each D_i consists of M samples: $D_i = \{x_{i1}, x_{i2}, \dots, x_{iM}\}$, where x_{ij} represents a data sample from point i. $y_{ij} \in \mathscr{Y}$ is the label for sample x_{ij}, indicating whether the traffic is benign or a specific type of attack.

Data from all points is combined into a single dataset:

$$D_{\text{central}} = \bigcup_{i=1}^{N} D_i \tag{1}$$

The learning objective is to train a model $f_\theta : \mathscr{X} \to \mathscr{Y}$, where θ is the model parameters, to minimize a loss function $\mathscr{L}$ over the combined dataset D_{central}. This can be formulated as:

$$\min_\theta \mathscr{L}(f_\theta(D_{\text{central}}), Y_{\text{central}}) = \min_\theta \sum_{i=1}^{N} \sum_{j=1}^{M} \ell(f_\theta(x_{ij}), y_{ij}) \tag{2}$$

Where ℓ is a loss function (e.g., cross-entropy) that quantifies the prediction error.

Few-Shot Learning Challenge: In few-shot learning, the available data for each class $c \in \mathscr{Y}$ is limited, meaning that for certain classes, the sample size M_c is small:

$$M_c \ll M$$

This scarcity of data introduces difficulty in accurately modeling rare classes, as the model f_θ lacks sufficient examples for effective generalization. Consequently, the model's ability to distinguish minority classes is constrained by the small sample size, leading to potential issues with high false alarm rates or missed detections for rare classes.

This challenge can be expressed as a limitation in minimizing $\mathscr{L}$:

$$\min_\theta \mathscr{L}(f_\theta(D_{\text{central}}), Y_{\text{central}}) \text{ subject to } M_c \ll M$$

b Federated Learning

The mathematical explanation of federated learning within network intrusion detection systems (NIDS):
θ_i: Local model parameters for client i θ_{global}: Global model parameters aggregated on the central server, representing a combined model learned across all clients $f_i(\theta_i)$: Local objective function (or loss function) for client i, which measures the prediction error on that client's data based on θ_i η: Learning rate, which controls the step size in the gradient descent process
Training Process in FL-NIDS

1. Local Training on Each Client:

Each client i trains a local model based on its data without sharing the data with other clients or the server. Given the current parameters θ_i^t at training round t, the model update is computed using gradient descent

$$\theta_i^{t+1} = \theta_i^t - \eta \nabla f_i(\theta_i^t) \tag{3}$$

Where: $\nabla f_i(\theta_i^t)$ is the gradient of the local objective function f_i with respect to the model parameters θ_i. This gradient, $\nabla f_i(\theta_i^t)$, represents how the model's parameters should be adjusted to reduce the local loss for client i. The update step $-\eta \nabla f_i(\theta_i^t)$ uses the learning rate η to control how much the model adjusts parameters in each iteration.

2. Aggregation on the Central Server:

After each client has performed its local update, the central server aggregates the updated parameters θ_i^{t+1} from each client to form the global model parameters $\theta_{\text{global}}^{t+1}$. A standard aggregation method is Federated Averaging (FedAvg), which calculates the weighted average of the client parameters:

$$\theta_{\text{global}}^{t+1} = \frac{1}{N} \sum_{i=1}^{N} \theta_i^{t+1} \tag{4}$$

Where N is the number of clients participating in training. The server broadcasts $\theta_{\text{global}}^{t+1}$ to all clients, and this serves as the starting model parameters for the next round.

3. Objective Function for Federated Learning:

The goal of Federated Learning is to minimize the overall objective function $F(\theta_{\text{global}})$, which is the sum of the local loss functions across all clients:

$$F(\theta_{\text{global}}) = \sum_{i=1}^{N} f_i(\theta_i) \tag{5}$$

Here: Each $f_i(\theta_i)$ represents the local loss on client i. The objective $F(\theta_{\text{global}})$ aims to find global parameters θ_{global} that minimize the cumulative error across all clients.

The mathematical structure of FL-NIDS in Federated Learning:

1. Each client i locally updates its parameters:

$$\theta_i^{t+1} = \theta_i^t - \eta \nabla f_i(\theta_i^t) \tag{6}$$

2. The central server aggregates the local parameters to update the global model:

$$\theta_{\text{global}}^{t+1} = \frac{1}{N} \sum_{i=1}^{N} \theta_i^{t+1} \tag{7}$$

3. The objective of FL-NIDS is to minimize:

$$F(\theta_{\text{global}}) = \sum_{i=1}^{N} f_i(\theta_i) \tag{8}$$

c Transfer Learning

Representation of Transfer Learning in NIDS:

Let $D_S = \{(x_i^S, y_i^S)\}$ represent the source domain dataset, where x_i^S are features and y_i^S are labels for samples in the source domain. Let $D_T = \{(x_i^T, y_i^T)\}$ represent the target domain dataset, with limited samples and rare classes.

In feature transfer, we aim to transfer learned feature representations from the source domain to the target domain. Let $f_{\theta_S}(x)$ be a feature extractor function, parameterized by θ_S, that is trained on the source domain:

$$\theta_S = \arg\min_{\theta} \sum_{(x_i^S, y_i^S) \in D_S} \ell(f_\theta(x_i^S), y_i^S) \tag{9}$$

The learned parameters θ_S are then adapted to the target domain by either fine-tuning or directly applying them, resulting in $f_{\theta_T}(x)$:

$$\theta_T = \theta_S + \Delta\theta \tag{10}$$

Where $\Delta\theta$ represents additional tuning or adjustments based on the target domain data D_T, allowing the model to adapt to the specific characteristics of the target environment.

In model transfer, a model trained on the source domain is fine-tuned or adapted for use in the target domain. If $F_{\theta_S}(x)$ is the model trained on the source domain data, then the objective is to adapt it to the target domain by minimizing the target loss function $\mathscr{L}_T$:

$$\theta_T = \arg\min_{\theta} \sum_{(x_i^T, y_i^T) \in D_T} \ell(F_\theta(x_i^T), y_i^T) \tag{11}$$

This process helps the model F_{θ_T} to learn from limited target domain data by leveraging pre-learned knowledge from the source domain.

The overall goal in transfer learning for NIDS is to minimize the loss on the target domain by starting with source domain knowledge:

$$\min_{\theta} \sum_{(x_i^T, y_i^T) \in D_T} \ell(F_{\theta_T}(x_i^T), y_i^T) + \lambda \sum_{(x_i^S, y_i^S) \in D_S} \ell(F_{\theta_S}(x_i^S), y_i^S)$$

Where λ is a balancing factor to control the influence of the source domain knowledge on the target domain learning.
